# Bivalent Inhibitor with Selectivity for Trimeric MMP-9 Amplifies Neutrophil Chemotaxis and Enables Functional Studies on MMP-9 Proteoforms

**DOI:** 10.3390/cells9071634

**Published:** 2020-07-07

**Authors:** Elisa Nuti, Armando Rossello, Doretta Cuffaro, Caterina Camodeca, Jens Van Bael, Dries van der Maat, Erik Martens, Pierre Fiten, Rafaela Vaz Sousa Pereira, Estefania Ugarte-Berzal, Mieke Gouwy, Ghislain Opdenakker, Jennifer Vandooren

**Affiliations:** 1 Department of Pharmacy, University of Pisa, Via Bonanno 6, 56126 Pisa, Italy; elisa.nuti@farm.unipi.it (E.N.); armando.rossello@farm.unipi.it (A.R.); doretta.cuffaro@farm.unipi.it (D.C.); caterina.camodeca@unipi.it (C.C.); 2 Laboratory of Immunobiology, Department of Microbiology, Immunology and Transplantation, Rega Institute for Medical Research, University of Leuven, KU Leuven, Herestraat 49-bus 1044, B-3000 Leuven, Belgium; jens.vanbael@kuleuven.be (J.V.B.); driesvandermaat@hotmail.com (D.v.d.M.); erik.martens@kuleuven.be (E.M.); pierre.fiten@kuleuven.be (P.F.); rafaela.pereira@kuleuven.be (R.V.S.P.); estefania.ugarteberzal@kuleuven.be (E.U.-B.); ghislain.opdenakker@kuleuven.be (G.O.); 3 Laboratory of Molecular Immunology, Department of Microbiology, Immunology and Transplantation, Rega Institute for Medical Research, University of Leuven, KU Leuven, Herestraat 49-bus 1044, B-3000 Leuven, Belgium; mieke.gouwy@kuleuven.be

**Keywords:** MMP-9, inflammation, endotoxemia, leukocytosis, chemotaxis, sepsis, acute inflammation, LPS, MMP, bivalent carboxylate inhibitor

## Abstract

A fundamental part of the immune response to infection or injury is leukocyte migration. Matrix metalloproteinases (MMPs) are a class of secreted or cell-bound endopeptidases, implicated in every step of the process of inflammatory cell migration. Hence, specific inhibition of MMPs is an interesting approach to control inflammation. We evaluated the potential of a bivalent carboxylate inhibitor to selectively inhibit the trimeric proteoform of MMP-9 and compared this with a corresponding monovalent inhibitor. The bivalent inhibitor efficiently inhibited trimeric MMP-9 (IC_50_ = 0.1 nM), with at least 500-fold selectivity for MMP-9 trimers over monomers. Surprisingly, in a mouse model for chemotaxis, the bivalent inhibitor amplified leukocyte influxes towards lipopolysaccharide-induced inflammation. We verified by microscopic and flow cytometry analysis increased amounts of neutrophils. In a mouse model for endotoxin shock, mice treated with the bivalent inhibitor had significantly increased levels of MMP-9 in plasma and lungs, indicative for increased inflammation. In conclusion, we propose a new role for MMP-9 trimers in tempering excessive neutrophil migration. In addition, we have identified a small molecule inhibitor with a high selectivity for the trimeric proteoform of MMP-9, which will allow further research on the functions of MMP-9 proteoforms.

## 1. Introduction

Neutrophils are the most abundant circulating leukocytes and the first cells to arrive at an inflammatory site. Here, they contribute to host defense and inflammation through release of pro-inflammatory and anti-microbial proteins, pre-stored in secretory vesicles for immediate release [1]. Next, the arrival of other inflammatory cells (e.g., monocytes/macrophages) further contributes to inflammation by de novo production and secretion of pro/anti-inflammatory proteins, by removal of particles through phagocytosis and by further orchestration of adaptive immunity [2,3]. Hence, interference with any of these steps may reduce inflammation and provides a valid strategy for the development of therapeutic drugs in the treatment of diseases characterized by excessive acute inflammation.

Neutrophil proteases mediate numerous steps of this inflammatory process. For example, they contribute to leukocyte recruitment and chemotaxis by shedding of selectins and integrins, degradation of cell junctional and extracellular matrix proteins and modulation of chemokine activity by limited proteolysis [4,5]. A major family of proteases implicated in inflammation are the matrix metalloproteinases (MMPs) [6]. These zinc-dependent endopeptidases cleave and modulate the activities of a range of chemokines, resulting in activation or inactivation of chemokines [5]. Whereas their roles in inflammation are now being recognized, MMPs were originally (± 20 years ago) studied as potent targets in cancer treatment, due to their ability to cleave different components of the extracellular matrix. Unfortunately, all early clinical trials with MMP inhibitors for cancer treatment failed due to a lack of inhibitor specificity and a general lack of knowledge on MMPs [7,8]. Due to the high structural homology between catalytic domains of members of the MMP-family, the design of specific small-molecule inhibitors is a challenging task, which has led to a search for alternative targeting strategies such as the design of allosteric inhibitors [9]. Furthermore, cancer treatment often involves long-term drug use and thus a better drug safety profile in contrast with shorter treatments such as those required for acute inflammations (e.g., sepsis syndromes) [7,10].

A particularly interesting MMP in inflammation is MMP-9. Large quantities of MMP-9 are found in specific and tertiary granules of human neutrophils, which are released at inflammatory sites [11] in the absence of the natural inhibitor tissue inhibitor of metalloproteinases-1 (TIMP-1) [12]. Neutrophils from MMP-9 knock-out mice are deficient in their ability to migrate to chemokine stimulation by granulocyte chemotactic protein-2 (GCP-2/CXCL6) [13]. Active MMP-9 cleaves several human chemokines, and results in inactivation of growth regulated protein alpha (GROα)/chemokine (C-X-C motif) ligand 1 (CXCL1), GROβ/CXCL2, platelet factor-4 (PF-4)/CXCL4, neutrophil activating peptide-2 (NAP-2)/CXCL7, monokine induced by gamma interferon (MIG)/CXCL9, interferon gamma-induced protein-10 (IP-10)/CXCL10 and stromal cell-derived factor-1-alpha (SDF-1α)/CXCL12, and potentiation of CXCL6 and interleukin-8 (IL-8)/CXCL8 at early stages of the inflammatory response [5]. The concept of collaborative actions of chemokines and MMP-9 is further strengthened by the discovery that MMP-9 (and MMP-2) activity at the blood-brain barrier promotes early chemokine-induced leukocyte migration [14]. Striking contributions of MMP-9 to acute inflammation are the induction of leukocytosis [15] and the detrimental effects in mouse models for endotoxemia. Upon induction of endotoxemia, neutrophils migrate from the bone marrow to the periphery, resulting in degranulation of MMP-8 (neutrophil collagenase) and MMP-9 into the circulation and in vital organs such as the lungs and liver [16]. Furthermore, MMP-9 knock-out mice are better protected against endotoxin shock than wild-type animals and mice treated with MMP-inhibitors (broad spectrum or combined against MMP-8, MMP-9 and a disintegrin and metalloproteinase17 (ADAM17)/tumor necrosis factor-α-converting enzyme (TACE)) are more likely to survive an endotoxin challenge or caecal ligation and puncture-induced sepsis [17,18,19]. Furthermore, MMP-9 is also able to cleave surface-associated CD40L on activated platelets, thereby controlling pulmonary accumulation of neutrophils in sepsis [20]. Hence, targeting of MMP-9 activity in such pathologies is a valid strategy.

MMP-9 has several unique features, which may be an opportunity for selective inhibition. For instance, proMMP-9 naturally occurs as monomers, multimers and covalent complexes with neutrophil gelatinase B-associated lipocalin (NGAL/lipocalin-2). The NGAL-proMMP-9 hetero-complex is a typical product of neutrophils and constitutes only a minor fraction of the total MMP-9 found in biological samples from healthy individuals (e.g., human serum). The major mass of MMP-9 are monomers and the second major fraction (>30–40%) in human and mouse tissues are multimeric. We recently discovered that these multimers contain stable ring-like entities consisting of three units of monomeric proMMP-9 [21]. Furthermore, we also found that endogenous protease inhibitors differentially regulate these MMP-9 proteoforms. Whereas trimeric MMP-9 is more efficiently inhibited by the local TIMP-1 compared to monomers, trimeric MMP-9 is able to partially overcome inhibition by the systemic inhibitor alpha-2-macroglobulin (α2M) [21,22]. 

To overcome the issues of inhibitor toxicity and selectivity, one approach is the design of bivalent inhibitors. This concept has been applied in several areas of medicinal chemistry, including the design of kinase inhibitors [23], cholinesterase inhibitors [24] and MMP-inhibitors [25,26,27]. Recently, carboxylate-based bivalent inhibitors more specific for MMP-9 were developed, with the aim to disrupt non-covalent MMP-9 dimerization and reduce cancer cell invasion which occurs through the MMP-9 hemopexin domain [26]. In this manuscript, we evaluated the ability of a bivalent carboxylate inhibitor (described as compound **7** by Nuti et al. [26]) to block the catalytic activity of stable MMP-9 trimers compared to monomers, and other proteases with significance in inflammation. Furthermore, we also investigated its effect on in vivo immune cell migration and its ability to mediate systemic inflammation in a mouse model for endotoxemia. The corresponding monovalent inhibitor **5** [26] was used for comparison. 

## 2. Materials and Methods

### 2.1. Proteins, Reagents and Buffers

Compounds **5** and **7** were synthesized as previously reported [26]. Full-length human proMMP-9 was produced by recombinant expression in Sf9 insect cells and purified by gelatin-Sepharose chromatography as previously described [28]. Stable full-length proMMP-9 monomers and trimers were separated by glycerol gradient ultracentrifugation as previously described [21,22]. ProMMP-9 mixture and the separated monomers and trimers were activated by incubation with the catalytic domain of MMP-3 (cat. No. 444217, Merck Millipore, Darmstadt, Germany) as previously described [28]. Activation of proMMP-9 was confirmed by a band shift of ±10 kDa, corresponding to the removal of the auto-inhibitory propeptide domain and by detection of gelatinolytic activity with a previously described gelatin degradation assay [29]. MMP-9 monomers and trimers purified from human neutrophils were purchased (Cat. No. 444,231 and 444232, Sigma-Aldrich, St. Louis, MO, USA). Examples of sample purities and activations can be found in Supplementary Figure S1. Recombinant human proMMP-2 (CHO cell-derived), proMMP-3 (NS0 cell-derived), proMMP-7 (NS0 cell-derived) and proMMP-8 (NS0 cell-derived) were purchased from R & D systems (Minneapolis, MN, USA), dissolved in assay buffer (150 mM NaCl, 5 mM CaCl_2_, 0.01% Tween-20, 50 mM Tris, pH 7.4) to a concentration of 100 µg/mL and activated by incubation with 1 mM p-aminophenylmercuric acetate for, respectively, 1h, 6h, 2h and 1h. Activation of MMPs was confirmed by a band shift of approximately 10 kDa, corresponding to the removal of the propeptide domain. MMP-14/MT1-MMP (NS0 cell-derived) was also purchased from R & D systems (Minneapolis, MN, USA) and activated by incubation with recombinant human furin (cat. No. 1503-SE-010, R & D systems, Minneapolis, MO, USA) in activation buffer (50 mM Tris, 1 mM CaCl_2_, 0.5% Brij-35, pH 9) for 1.5 h and activity was tested in assay buffer (50 mM Tris, 3 mM CaCl_2_, 1 µM ZnCl_2_, pH 8.5). Active human neutrophil elastase (NE) was purchased from Abcam (Cambridge, UK) and for activity assays of NE the assay buffer was replaced by 200 mM Tris, pH 8.8. ADAM17/TACE (Sf 21 (baculovirus)-derived, R & D systems, Minneapolis, MO, USA) was diluted and evaluated in 25 mM Tris, 2.5 μM ZnCl_2_, 0.005% Brij-35, pH 9.0. All stocks of the compounds were evaluated for the presence of endotoxin using the EndoZyme II assay (Hyglos bioMérieux, Bernried am Starnberger See, Germany). Endotoxin levels in all concentrated stocks (2.5 mg/mL) were below the accepted limit of 0.5 EU/mL.

### 2.2. Gelatin and Peptide Degradation Assays

To determine the inhibition of gelatinolytic activity, we used a previously optimized gelatin degradation assay [29]. Activated MMP-9, activated MMP-2, neutrophil elastase or supernatants of stimulated neutrophils, were incubated with different concentrations of inhibitors and incubated for 30 min at 37 °C. Next, dye-quenched gelatin (DQ™-gelatin, Invitrogen, Carlsbad, CA, USA) was added at a final concentration of 2.5 µg/mL and the increase in fluorescence over time was measured with the CLARIOstar microplate reader (BMG Labtech, Ortenberg, Germany). For other MMPs, the OmniMMP substrate peptide (Mca-PLGL-Dpa-AR-NH_2_, cat. no. BML-P126-0001, Enzo Life Sciences, Farmingdale, NY, USA) was used at a final concentration of 2.5 µg/mL. For ADAM17 the fluorogenic peptide Mca-PLAQAV-Dpa-RSSSR-NH_2_ (ES003, R & D systems, Minneapolis, MO, USA) and for neutrophil elastase the fluorogenic substrate MeOSuc-AAPV-AMC (cat. no. 324740, Merck Millipore, Darmstadt, Germany) were used. Proteolytic activity was determined by linear regression of the fluorescence curve and the percentage inhibition was calculated through comparison with the positive control (no inhibitors). When testing supernatants from human neutrophils, the assay was performed in the presence of 20 µM neutrophil elastase inhibitor (Elastase Inhibitor IV, Calbiochem, Darmstadt, Germany).

### 2.3. Isolation, Degranulation and Chemotaxis of Human Neutrophils

Neutrophils were isolated from fresh blood from healthy donors, via density gradient centrifugation as described [30]. To obtain neutrophil degranulate, neutrophils were suspended in degranulation buffer (120 mM NaCl, 15 mM CaCl_2_, 20 mM Tris/HCl pH 7.5) at a concentration of 10^7^ cells/mL and degranulation was induced by incubating neutrophils with N-formyl-methionyl-leucyl-phenylalanine (fMLF) (final concentration 0.5 µM) for 20 min at 37 °C as previously described [18]. Next, the supernatant was collected by centrifugation. For fresh blood donations, all subjects gave written informed consent in accordance with the Declaration of Helsinki.

### 2.4. Compound Toxicity and Stability Testing

Toxicity of compounds **5** and **7** was evaluated in vitro on the U-87 cell line. Briefly, U-87 cells were seeded in 96-well plates (cat. No. 92096, TPP Techno Plastic Products, Trasadingen, Switzerland) at 2000 cells/well in DMEM FluoroBrite containing 10% FBS and incubated for 24 h. Cells were washed and further grown in DMEM FluoroBrite with 5% KnockOut™ Serum Replacement (Gibco, Waltham, MA, USA). Compounds **5** and **7** were added to the wells in a 1:2 dilution series starting at 20 µM and ending at 0.16 µM. To evaluate the effect of the compound on cell growth, confluence was determined every hour for 17h using the Incucyte^®^ S3 Live-Cell Analysis System (Sartorius, Göttingen, Germany). Next, cells were stained with the LIVE/DEAD™ Viability/Cytotoxicity Kit for mammalian cells (Invitrogen, Carlsbad, CA, USA; cat. # L3224) and cell viability was evaluated. Data were normalized to the mean confluence of wells containing no inhibitors. To measure the stability of the compounds, fresh blood was collected from heathy mice by cardiac puncture with a heparin-coated needle and syringe and 30UI/mL heparin was added. Next, compound **5**, compound **7** or vehicle (DMSO) was added to the blood at a final concentration of 500 µM and incubated at 37 °C. At indicated time periods, samples were taken, diluted ½ in phosphate-buffered saline (PBS) and centrifugated at 500× *g* for 5 min to collect plasma. The cell pellet was washed twice with PBS and cells were lysed in radioimmunoprecipitation assay buffer (RIPA) buffer (25 mM Tris, 150 mM NaCl, 1% NP-40, 1% Sodium Deoxycholate, 0.1% SDS, pH 7.6). To determine the remaining inhibitory activity of the compounds, the plasma and cell lysates were incubated with active recombinant MMP-9 (0.5 nM) and the remaining MMP-9 activity was measured using the OmniMMP substrate peptide. Background inhibition was subtracted and results were expressed as percentage inhibition.

### 2.5. Mouse Air Pouch Model

Air pouches were established by injecting C57BL/6 mice on dorsal sites with 3 mL filtered air (0.20 μm filter) on days 0 and 3. On day 6, test samples were injected in a total volume of 1 mL and at the indicated amount. After 4 h, mice were euthanized and the exudates of the pouches were collected after injection of 1 mL pyrogen-free PBS containing 20 U/mL heparin and 2% KnockOut™ Serum Replacement (Gibco, Waltham, MA, USA), and followed by 30 sec of gentle massage [31]. This procedure was repeated twice with 2 mL buffer. Next, the pouch fluid was centrifuged (10 min, 500× *g*) and supernatant and cell pellets were separated for further analysis. Data were collected during three independent experiments with 3–4 mice per group. All procedures were conducted in accordance with protocols approved by the local Ethics Committee (License number P033/2018, Belgium).

### 2.6. Cytospin Preparation and Manual Cell Counting

For microscopic evaluation of cells derived from air pouches, 75 × 10^3^ cells were deposited evenly onto a glass slide by centrifugation using a Shandon Cytospin 2 apparatus (Thermo Shandon, Pittsburgh, PA, USA). Next, the preparations were stained with Hemacolor (Merck Chemicals, Darmstadt, Germany). Cells were divided into 4 categories; neutrophils, monocytes/macrophages, lymphocytes and other cells. For each condition, three times 100 cells were counted and the average was used as a final value.

### 2.7. Flow Cytometry

Approximately 10^6^ cells were incubated for 15 min with the Fc-receptor-blocking antibodies anti-CD16/anti-CD32 (BD Biosciences Pharmingen, San Diego, CA, USA) and with a Zombie Aqua™ viability dye (BioLegend, San Diego, CA, USA). After washing with PBS + 2% fetal calf serum (FCS), the cells were stained for 30 min with the fluorescein isothiocyanate (FITC)-conjugated anti-CD3 (cat. No. 11-0031-81, eBioscience, San Diego, CA, USA), phycoerythrin (PE)-conjugated anti-Ly6G (cat. No. 12-9668-82, eBioscience, San Diego, CA, USA), allophycocyanin (APC)-conjugated anti-F4/80 (cat. No. 17-4801-80, eBioscience, San Diego, CA, USA) and PERCP-Cy5.5-conjugated anti-CD11b (cat. No. 45-0112-80, eBioscience, San Diego, CA, USA). Next, cells were washed twice and fixed with 0.37% formaldehyde in PBS. Cells were analyzed on a BD LSR Fortessa X20 with DIVA software (BD, Franklin Lakes, NJ, USA, v9.0). Results were further analyzed with the FlowJo software (BD, Franklin Lakes, NJ, USA, v10.0). Flow cytometry plots showing the gating strategy for cellular identification are depicted in Supplementary Figure S2. 

### 2.8. Mouse Endotoxemia Model

Endotoxemia was induced in 8-week old C57BL/6 mice by intraperitoneal (i.p.) injection of lipopolysaccharides (LPS) (*Escherichia coli* 0111:B4, cat. no. 4391, Sigma-Aldrich, St. Louis, MO, USA ) at 10 mg/kg. Control mice were injected with an equal volume of vehicle (pyrogen-free phosphate-buffered saline). A volume of 500 µl compound **5** or **7**, diluted in PBS with 2% DMSO (2 mg/mL), was injected i.p. at the same time as the LPS injection (± 50 mg/kg). In animals that did not receive test compounds, this volume was replaced with 2% DMSO in PBS. After induction of endotoxemia, progression of the disease was monitored by hourly evaluation of mouse body temperature (rectal probe) and disease scoring by use of the mouse sepsis score (MSS) as described [32]. When the MSS score was above 21, humane endpoints were reached and mice were sacrificed. Blood was collected by cardiac puncture with a heparin-coated needle and syringe, and immediately processed by centrifugation (2000× *g* for 10 min at 4 °C). The supernatant (plasma) was collected and stored for further analysis. Mice were dissected and organs were stored for further analysis. Bone marrow cells were collected from femora by flushing the medullary cavity with PBS and after centrifugation the cell pellet was immediately processed for downstream analysis. All procedures were conducted in accordance with protocols approved by the local ethics committee (project number P128/2019, KU Leuven, Belgium).

### 2.9. Protein Extraction and Analysis

Tissues were homogenized and proteins extracted with a Precellys lysing kit (Bertin Technologies, Rockville, MD, USA). Briefly, tissues were placed in hard tissue homogenizing CK28 tubes (Bertin Technologies, Rockville, MD, USA). Then, 600 µL of RIPA buffer was added to all tubes and homogenization was done using the Precellys^®^24 (Bertin Technologies, Rockville, MD, USA). To precipitate all tissue debris, the tubes were centrifuged at 20,800× *g* and 4 °C for 15 min. Supernatant, containing soluble proteins, was collected and used for further analysis. Total protein content of all samples was determined by using a standard Bradford assay (Bio-Rad, Hercules, CA, USA). For Western-blot analysis, samples were chemically reduced and the indicated amount of proteins were separated on 16% Novex Tris-glycine gels in a mini gel tank as instructed by the supplier (Invitrogen, Carlsbad, CA, USA). Proteins were transferred to PVDF membranes using the Trans-Blot Turbo Transfer System with associated materials and protocols (Biorad, Hercules, CA, USA). Next, membranes were blocked for 1 h in 5% BSA with TBST buffer (150 mM NaCl, 0.1% Tween 20, 50 mM Tris, pH 7.5). Membranes were incubated overnight with anti-MMP-9 (AF909, R & D systems, Minneapolis, MO, USA) or anti-tubulin (MA5-16308-HRP, Invitrogen, Carlsbad, CA, USA) antibodies. After washing, the blot was incubated with peroxidase-conjugated anti-goat IgG (PI-9500, Vector Labs, Burlingame, CA, USA) or anti-mouse IgG (115-035-071, Jackson ImmunoResearch, PA, USA) for 1 h at room temperature. Finally, Western blots were imaged using the Vilber Lourmat Fusion system (Labtech International, Heathfield, TN, USA) and Pierce ECL Western Blotting Substrate (Thermo Fisher Scientific, Waltham, MA, USA). 

### 2.10. Statistics

Data were analyzed using the GraphPad Prism software (GraphPad Software, San Diego, CA, USA, Prism 8) and biochemical dose-response curves were fitted as indicated. Data derived from mouse experiments were first analyzed for outliers using the ROUT method (Q = 1%) and data distribution was tested using the D’Agostino and Pearson normality test. Statistical differences were tested either by ordinary one-way ANOVA with Holm-Sidak’s multiple comparisons test (normal distributed data) or Kruskal–Wallis test with Dunn’s multiple comparisons test (no normal distribution or low sample number).

## 3. Results

### 3.1. Bivalent Carboxylate Inhibitor Efficiently Inhibits Homotrimeric MMP-9

**Figure 1:**
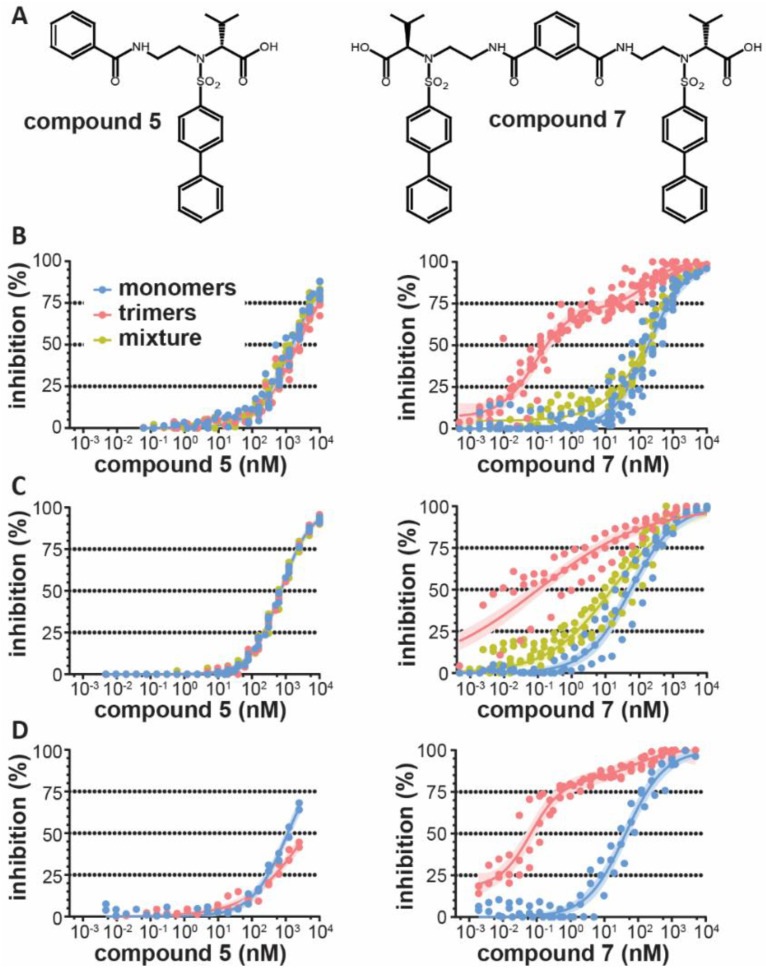
Inhibition of matrix metalloproteinase (MMP)-9-mediated proteolysis by monovalent and bivalent carboxylate inhibitors. (**A**) Chemical structure of the monovalent (compound **5**, left) and the corresponding bivalent (compound **7**, right) carboxylate inhibitors [26]; (**B**) inhibition of gelatinolytic activity of recombinant human MMP-9 monomers, trimers and their mixture (± 70% monomers and ± 30% trimers) by the monovalent inhibitor (left panel) and bivalent inhibitor (right panel); (**C**) inhibition of peptidolytic activity of recombinant human MMP-9 monomers, trimers and their mixture (± 70% monomers and ± 30% trimers) by the monovalent inhibitor (left panel) and bivalent inhibitor (right panel); (**D**) inhibition of gelatinolytic activity of human neutrophil-derived MMP-9 monomers or trimers by the monovalent inhibitor (left panel) and bivalent inhibitor (right panel). Data were fitted with either a standard or biphasic dose-response fit, as indicated in Table 1. Surface area fill represents the 95% confidence interval of the fit.

Considering the close proximity of MMP-9 active sites in the homotrimeric MMP-9 proteoform [21], we hypothesized that bivalent inhibitors might be more potent at inhibiting the trimeric population of MMP-9 than monovalent inhibitors. Both inhibitors (previously reported as compound **5** and compound **7** [26], Figure 1A) are zinc-chelating ligands bearing a carboxylate as zinc-binding group, as shown by previous X-ray crystallographic studies [33]. We first evaluated the ability of the monovalent and bivalent carboxylate inhibitors to inhibit gelatinolysis by recombinant human monomeric and trimeric MMP-9. A first observation was that the bivalent inhibitor had an overall improved ability to inhibit MMP-9 of all proteoforms (IC_50_ of 1407 nM (compound **5**) versus 182 nM (compound **7**) for MMP-9 mixtures, Table 1). Furthermore, whereas the monovalent inhibitor did not differentially affect monomeric and trimeric MMP-9, the bivalent inhibitor was over 1000-fold more potent at inhibiting gelatinolysis by trimeric MMP-9. The dose-response curve followed a biphasic course for MMP-9 trimers (Figure 1B) and the IC_50_-value of the bivalent inhibitor for trimeric MMP-9 was calculated at 0.18 nM, which is lower than most existing MMP-9 inhibitors [10]. Next, we repeated the experiment with a small fluorogenic peptide substrate to exclude effects due to exosite binding. This also resulted in a 560-fold lower IC_50_-value for the “bivalent” inhibitor against trimeric MMP-9, with a low IC_50_-value of 0.1 nM (Figure 1C and Table 1). Finally, we also repeated these experiments with MMP-9 monomers and trimers purified from human neutrophils (Figure 1D and Table 1). Again, the bivalent inhibitor overall performed better and discriminated between monomeric and trimeric MMP-9 (650-fold difference). The low IC_50_-value for the bivalent inhibitor against MMP-9 trimers was again confirmed to be considerably low (IC_50_ = 0.066 nM). Therefore, we concluded that the bivalent carboxylate inhibitor (compound **7**) most effectively shuts down the proteolytic activity of the trimeric population of MMP-9.

### 3.2. Bivalent Inhibitor Is Most Potent Against Trimeric MMP-9, Out of Several Leukocyte-Derived Proteases

**Figure 2:**
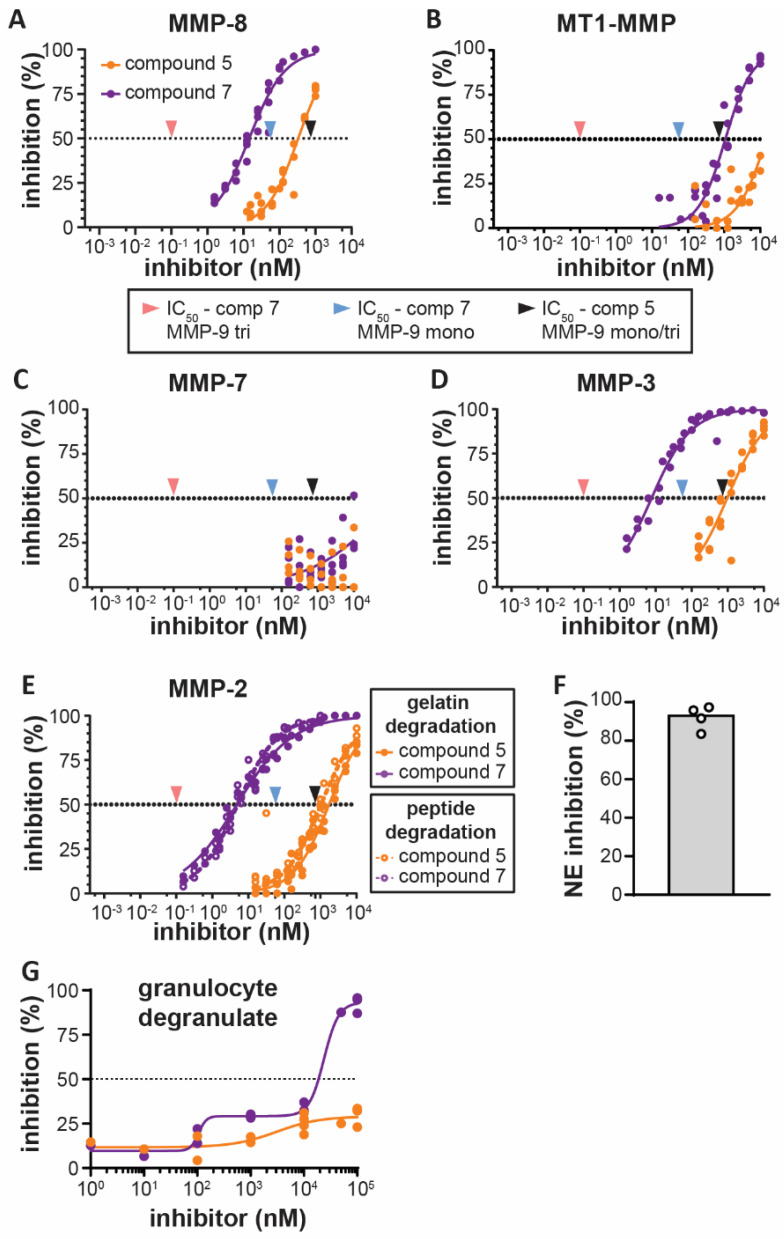
Inhibition of other proteases by monovalent and bivalent carboxylate inhibitors. (**A**) Inhibition of proteolytic activity of MMP-8 by the monovalent inhibitor (orange) and bivalent inhibitor (purple). Arrows indicate the IC_50_-values for the inhibition of MMP-9 trimers by the bivalent inhibitor (red), inhibition of MMP-9 monomers by the bivalent inhibitor (blue) and inhibition of MMP-9 monomers and trimers with the monovalent inhibitor (black); (**B**) inhibition of proteolytic activity of membrane-type-1-MMP (MT1-MMP)/MMP-14 by the monovalent inhibitor (orange) and bivalent inhibitor (purple); (**C**) inhibition of proteolytic activity of MMP-7 by the monovalent inhibitor (orange) and bivalent inhibitor (purple); (**D**) inhibition of proteolytic activity of MMP-3 by the monovalent inhibitor (orange) and bivalent inhibitor (purple); (**E**) inhibition of proteolytic activity of MMP-2 by the monovalent inhibitor (orange) and bivalent inhibitor (purple), as tested with a fluorogenic gelatin substrate (closed circles) and a fluorogenic peptide substrate (open circles); (**F**) percentage of gelatinolytic activity in the secretions of fMLF stimulated human granulocytes inhibited by a neutrophil elastase inhibitor (20 µM). Granulocyte secretions from four different blood donors were analyzed (*n* = 4). Each data point is indicated by a circle; (**G**) inhibition of non-neutrophil elastase-derived gelatinolytic activity in secretions of fMLF stimulated human granulocytes by compound **5** (orange) and compound **7** (purple). Data were fitted with a dose-response curve and all tests were done in the presence of 20 µM neutrophil elastase inhibitor. Each data point represents a different blood donor (*n* = 4).

To evaluate the specificity of the inhibitors in the context of inflammation, we next investigated the effect of the monovalent and bivalent carboxylate inhibitors on other proteases secreted by infiltrating leukocytes or epithelial cells. The main proteases found in secretory vesicles of neutrophils are matrix metalloproteinases (MMP-9 and MMP-8), a disintegrin and metalloproteinases (ADAMs) (such as ADAM-17) [34,35] and serine proteases (neutrophil elastase, cathepsin G and proteinase-3). As expected, no inhibitory effect was seen against serine proteases, here exemplified by neutrophil elastase (Table 2). ADAM-17, also known as TACE, a metalloprotease and an important regulator of tumor necrosis factor (TNF) signaling [6,36], was not inhibited by compound **5** nor compound **7**. In contrast, MMP-8 (also called neutrophil collagenase) was sensitive to inhibition by both compounds (Figure 2A and Table 2). However, inhibition of MMP-8 by compound **7** was still more than 100-fold less than for trimeric MMP-9. Hence, the action of the two tested inhibitors seemed mostly limited to MMPs. Recently, it was shown that LPS-stimulated macrophages release two collagenases (MMP-8 and MMP-13) and membrane-type-1-MMP (MT1-MMP) or MMP-14 [37]. Both compounds **5** and **7** were able to inhibit MMP-14-mediated proteolysis, albeit only at high concentrations (IC_50_ > 1 µM) (Figure 2B and Table 2). Furthermore, in classically activated (M1) macrophages, upregulated mRNA expression of several MMPs with other substrate specificities are reported, including MMP-3 (stromelysin-1) and MMP-7 (matrilysin) [38]. Indeed, MMP-7, also produced in epithelium of tissues such as lung, liver and breast, has been associated with influx of inflammatory cells to sites of tissue injury through cleavage of syndecan-1 [39]. Similarly, MMP-3 contributes to tissue damage in neutrophil-mediated pathologies, including arthritis [40] and acute pulmonary inflammation [41]. Whereas MMP-7 was only weakly (IC_50_-value >10 µM) inhibited by both of our inhibitors (Figure 2C and Table 2), MMP-3 was inhibited by both compounds, with a low IC_50_-value for the bivalent inhibitor (7.7 nM), although still approximately 70 times higher than for trimeric MMP-9 (Figure 2D and Table 2). Finally, we also tested inhibition of MMP-2. MMP-2 is a gelatinase (gelatinase-A), however, it is less implicated in inflammation than MMP-9. As expected, IC_50_-values for the dimeric inhibitor against MMP-2 were most similar to MMP-9 (IC_50_-value compound **7** = ± 5 nM) and this was confirmed with a gelatin substrate and a peptide substrate (Figure 2E and Table 2). In conclusion, both inhibitors were able to inhibit several MMPs. The monovalent inhibitor (compound **5**) was most active against MMP-8 (IC_50_ = 331 nM), followed by MMP-9 monomers and trimers (IC_50_ = ± 720 nM). However, the bivalent inhibitor (compound **7**) was most effective against trimeric MMP-9 (IC_50_ = 0.1 nM), followed by MMP-2 (another gelatinase, IC_50_ = 5 nM), MMP-3 (IC_50_ = 7.7 nM), MMP-8 (IC_50_ = 14.5 nM) and MMP-9 monomers (IC_50_ = 56 nM). Overall, the inhibitory capacity of the bivalent carboxylate inhibitor on the trimeric proteoform of MMP-9 was at least 50-fold higher than for the tested MMPs.

### 3.3. Bivalent Inhibitor Most Efficiently Inhibits Gelatinolysis in Secretions of Stimulated Human Granulocytes

We next investigated the ability of compounds **5** and **7** to inhibit gelatinolysis in secretions of stimulated human granulocytes. In line with our previous studies [16], human granulocytes stimulated with fMLF contained considerable gelatinolytic activity of which 84–97% was inhibited by addition of a neutrophil elastase inhibitor (depending on the donor, Figure 2F) and the remaining signal was sensitive to metalloprotease-inhibitors. Both compounds **5** and **7** were able to further reduce metalloproteinase-derived gelatinolytic activity (Figure 2G). However, the dimeric inhibitor was considerably more potent than the monovalent inhibitor in this read-out. Whereas the monomeric inhibitor was only able to inhibit approximately 30% of the signal at the highest dose of 100 µM, the dimeric inhibitor reached almost complete inhibition at this concentration. The dimeric inhibitor showed a biphasic inhibition profile, reaching a first inhibition plateau when inhibiting approximately 30% of the signal around a concentration of 1 µM.

### 3.4. In Vitro Toxicity and Ex Vivo Stability

**Figure 3:**
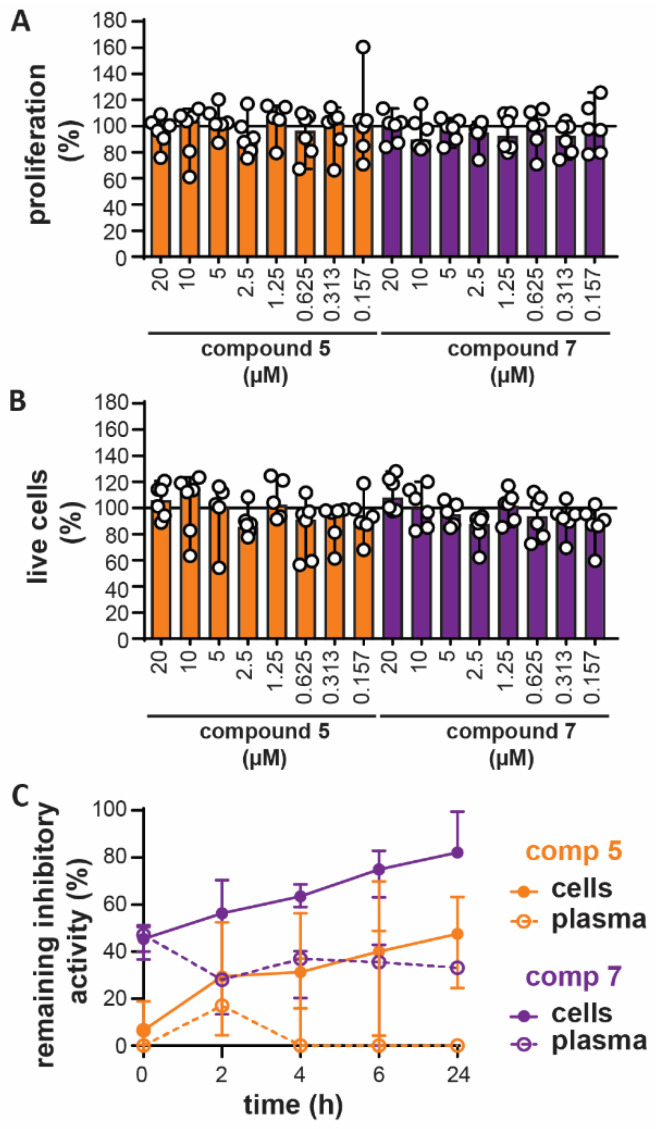
In vitro toxicity and ex vivo stability of compounds **5** and **7**. (**A**) Effect of compounds **5** and **7** on the proliferation of U-87 cells. Data represent 17 h of growth in the presence of indicated concentrations of the compounds and in comparison with cells grown without compounds (*n* = 5–6); (**B**) effect of compounds **5** and **7** on the cell viability of U-87 cells grown for 17 h in the presence of indicated concentrations of the compounds and in comparison with cells grown without compounds. (*n* = 5–6); (**C**) evaluation of the ex vivo stability of the compounds by measurement of the remaining MMP-9 inhibitory activity after addition of the compounds to mouse blood for the indicated period. Inhibitory activity present in the plasma (open circles, dotted lines) and cell lysates (closed circles, solid lines) was evaluated (*n* = 3).

Prior to testing of the compounds in animal models, we first excluded major toxic effects or low product stability. Up to a concentration of 20 µM, compounds **5** and **7** did not reduce proliferation of the U-87 cell line (Figure 3A), nor did they induce cellular toxicity (Figure 3B). Furthermore, when added to freshly isolated mouse blood, inhibitory activity of compounds **5** and **7** remained detectable up to 24h later (Figure 3C). Interestingly, over time, the inhibitory activity shifted from plasma towards the cell lysates, indicating that the compounds might be subject to cellular adhesion or uptake.

### 3.5. Bivalent Inhibitor Amplifies In Vivo Chemotaxis of Neutrophils Towards LPS injection

**Figure 4:**
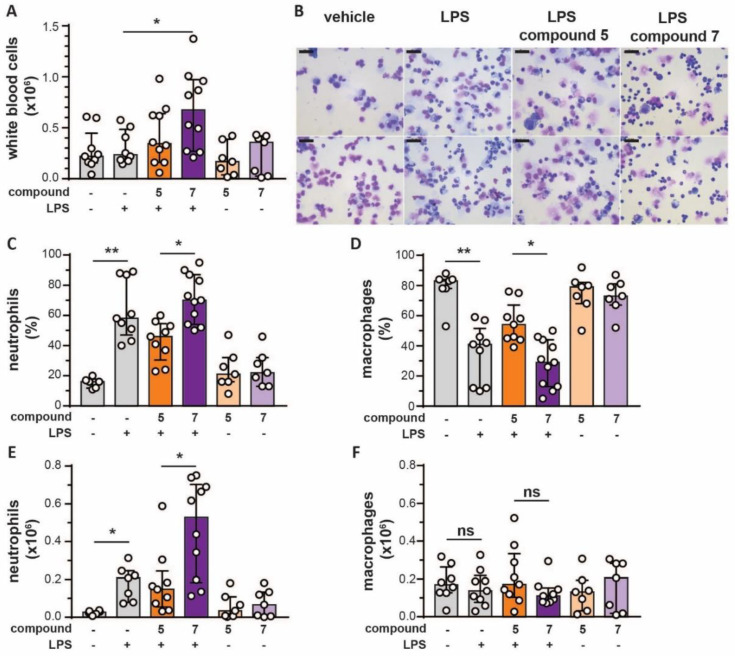
Effects of compounds **5** and **7** on in vivo chemotaxis of neutrophils and macrophages. (**A**) Total amount of white blood cells migrated to subdermal air pouches containing compound **5** (5 µg) and compound **7** (5 µg) in the presence (+) or absence (-) of lipopolysaccharides (LPS) (2 µg), 4 h after injection; (**B**) representative microscopy images of cytospin preparations from cells obtained from air pouches. Two images are shown per condition. Size bars represent 20 µm; (**C**) percentages of neutrophils present in air pouches as determined by differential cell counting of cytospin preparations; (**D**) percentages macrophages present in air pouches as determined by differential cell counting of cytospin preparations; (**E**) total amounts of neutrophils recruited to air pouches; (**F**) total amounts of macrophages recruited to air pouches. Each data point represents a single mouse (*n* = 7–10). * *p* < 0.05, ** *p* < 0.01. Bars represent median values and error bars represent interquartile ranges. Data were pooled from three independent experiments. +/- signs indicate, respectively, the presence or absence of LPS (2 µg).

**Figure 5:**
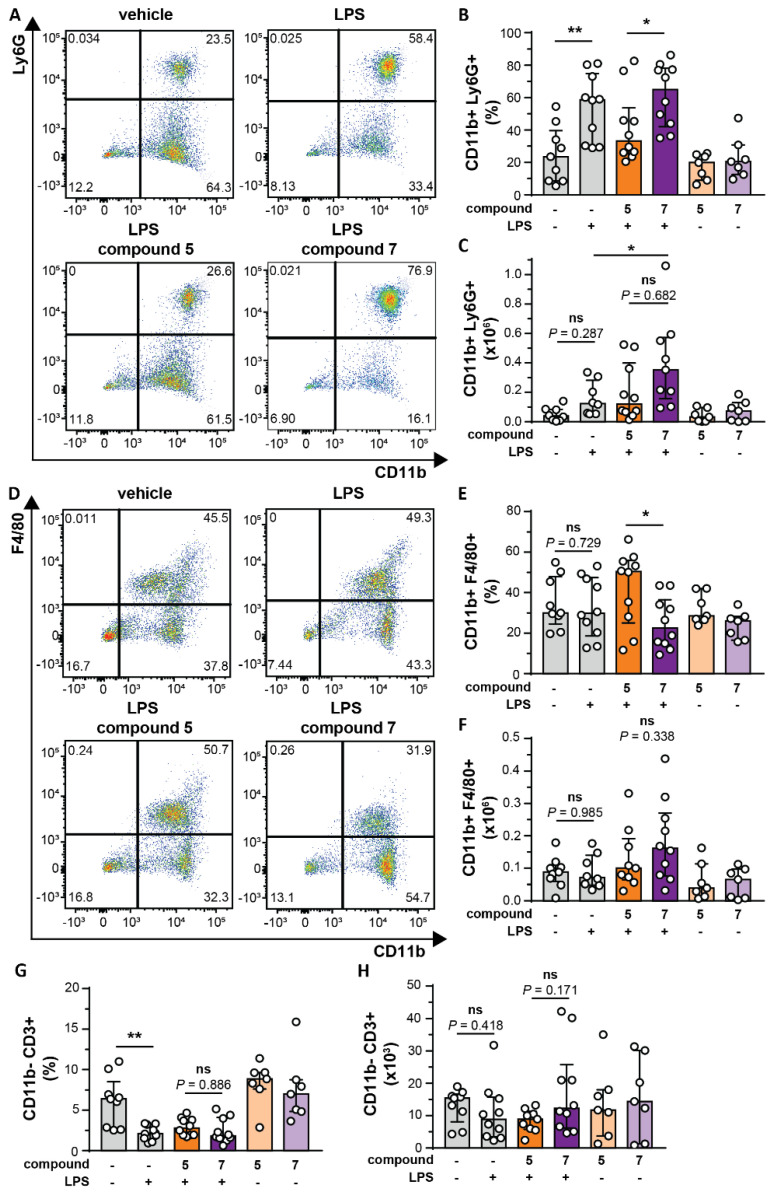
Effects of compounds **5** and **7** on in vivo chemotaxis of CD11b^+^ Ly6G^+^, CD11b^+^ F4/80^+^ and CD11b^-^ CD3^+^ cells. (**A**) Neutrophil content of pouches from mice given a vehicle injection (top left), an LPS injection (top right), an LPS injection and compound **5** (bottom left) or an LPS injection and compound **7** (bottom right). Flow cytometry plots represent the distribution of live cells based on surface CD11b and Ly6G staining; (**B**) analysis of the percentage of neutrophils (CD11b+Ly6G+) recruited to air pouches; (**C**) total number of neutrophils (CD11b+Ly6G+) migrated to subdermal air pouches; (**D**) macrophage content of pouches from mice given a vehicle injection (top left), an LPS injection (top right), an LPS injection and compound **5** (bottom left) or an LPS injection and compound **7** (bottom right). Flow cytometry plots represent the distribution of live cells based on surface CD11b and F4/80 staining; (**E**) analysis of the percentage of macrophages (CD11b+F4/80+) recruited to air pouches. (**F**) Total number of macrophages (CD11b+F4/80+) migrated to subdermal air pouches. (**G**) Analysis of the percentage of T-cells (CD11b-CD3+) recruited to air pouches. (**H**) Total number of T-cells (CD11b-CD3+) migrated to subdermal air pouches. Air pouches contain compound **5** (5 µg) and compound **7** (5 µg) in the presence or absence of LPS (2 µg). Each data point represents a single mouse (*n* = 7–10). *p < 0.05, **p < 0.01. Bars represent mean values. Data were pooled from three independent experiments. +/- signs indicate, respectively, the presence or absence of LPS (2 µg).

Since several studies have associated MMP-8 and MMP-9 with neutrophil recruitment [13,42,43], we wondered how in vivo inhibition by chemical compound **5**, and in particular by our MMP-9 trimer-specific compound **7** might affect neutrophil chemotaxis. As a result that neutrophils are the main producers of TIMP-1-free MMP-9 [12] and respond most quickly to inflammatory cues, we studied short-term in vivo parameters as a read-out of the effects of the new inhibitors. As a model for in vivo chemotaxis, *E. coli*-derived LPS, with or without inhibitors, were injected into dorsal air pouches generated in C57BL/6 mice [31]. Surprisingly, after four hours, the total amount of white blood cells recruited to the pouches with LPS and treated with compound **7** showed a significant increase compared to LPS alone (Figure 4A), suggesting that compound **7** aids white blood migration towards an inflammatory trigger. Microscopic evaluation of the cells (Figure 4B) and manual counting revealed a significant increase in the percentages of neutrophils in pouches treated with LPS and a significant difference between LPS-pouches treated with compound **5** and compound **7** (Figure 4C). In addition, this also resulted in relatively lower levels of macrophages (Figure 4D). Absolute numbers of neutrophils recruited to the pouches were also significantly increased in the LPS condition and a significant difference was seen between LPS-pouches treated with compound **5** and compound **7** (Figure 4E). No differences were seen in the absolute numbers of macrophages. To further confirm our finding, we also performed flow cytometry analysis. Indeed, the percentage of neutrophils (CD11b+Ly6G+ cells) was significantly increased in the LPS condition and a significant difference existed between pouches treated with compounds **5** and **7** (Figure 5A,B). Although these differences were reflected in the absolute numbers, the only significant difference was between the LPS condition and LPS with compound **7** (Figure 5C). When analyzing macrophages (CD11b+F4/80+ cells), again a relative and significant difference was seen between compounds **5** and **7** in the presence of LPS (Figure 5D,E), which did not translate in significant differences in absolute numbers (Figure 5F). Finally, no significant differences were seen in the percentages (Figure 5G) and absolute numbers (Figure 5H) of T-cells (CD11b-CD3+) in the presence of the tested compounds. As a conclusion, by the comparison of a monovalent versus bivalent carboxylate inhibitor, having high selectivity for MMP-9 trimers, we were able for the first time to demonstrate differential effects in vivo.

### 3.6. Bivalent Inhibitor Does Not Differentially Affect Disease Scores in a Mouse Endotoxemia Model

**Figure 6:**
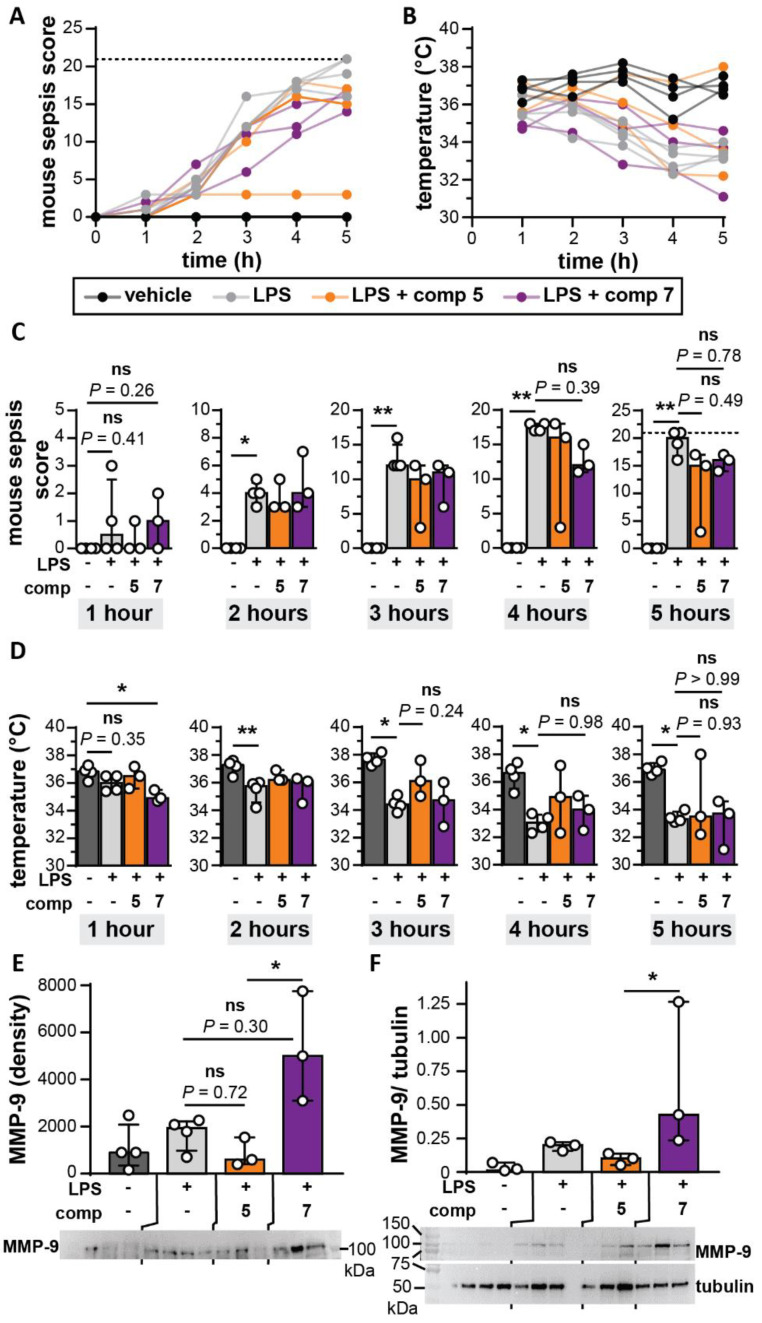
Endotoxemia mouse model. (**A**) Mouse sepsis score and (**B**) body temperature of control animals (vehicle, black), mice receiving 10 mg/kg LPS (grey), mice receiving 10 mg/kg LPS and 1 mg compound **5** (orange) and mice receiving 10 mg/kg LPS and 1 mg compound **7** (orange); (**C**) comparison of mouse sepsis score and (**D**) body temperature, each hour after LPS/compound injection. (**E**) Western-blot analysis of total MMP-9 found in mouse plasma, 5 h after LPS/compound administration. (**F**) Western-blot analysis of total MMP-9 found in mouse lungs, 5 h after LPS/compound administration and relative to a loading control (tubulin). Each data point represents a single mouse (*n* = 3–4). *p < 0.05, **p < 0.01. Bars represent median values. Full Western-blot images are available in Supplementary Figure S3. +/- signs indicate, respectively, the presence or absence of LPS (2 µg).

Since inhibitors with limited MMP specificity have been found to improve the outcome in mouse models for sepsis, we also evaluated the effects of compound **5** and **7** in a mouse model for endotoxemia [18,19,44]. Endotoxemia was induced by i.p. injection of 10 mg/kg LPS and our compounds were administered i.p. simultaneously. A compound dose of 50 mg/kg was chosen based on previous studies with small-molecule MMP inhibitors [45,46]. Disease progression was monitored by scoring several parameters of disease development (mouse sepsis score, MSS [32]) (Figure 6A) and by measuring body temperature (Figure 6B). All mice that received LPS developed sepsis-like symptoms, except for one animal receiving compound **5**, which developed milder symptoms. Although no significant differences were found in disease scoring and temperature (Figure 6C,D), it appeared that compound **7** induced an early and significant drop in mouse body temperature and an (although not significant) increase in disease score, pointing towards an early effect of compound **7**. After five hours, some mice reached the humane endpoint (MSS >21), which resulted in termination of the experiment and collection of tissue samples from all animals. Since in previous studies, it was shown that induction of endotoxemia results in an increase of MMP-9 in blood and in organs affected by systemic inflammations [16,47], we also investigated the presence of MMP-9. As expected, higher levels of MMP-9 were found in plasma and lungs of endotoxemic mice (Figure 6E,F and Figure S3). Interestingly, the effect of compound **7** seemed to further induce the presence of MMP-9 and was found to be significantly different from the effect of compound **5**.

## 4. Discussion

**Figure 7:**
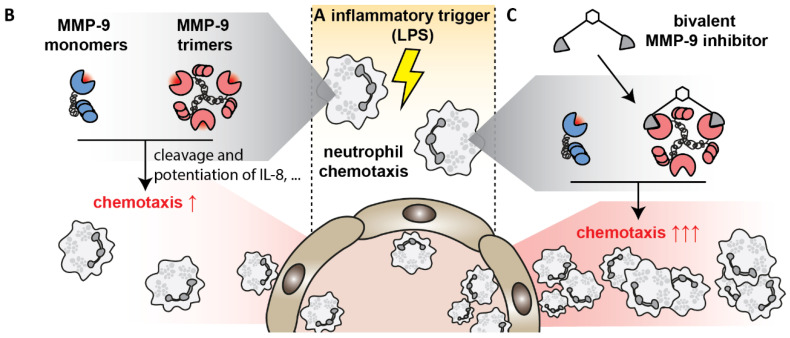
Study overview. (**A**) At early stages in infections or inflammations, neutrophils migrate towards an inflammatory stimulus (e.g., *E. coli* LPS) and release proteolytic or bactericidal proteins, including MMP-9; (**B**) through actions such as the cleavage and potentiation of the early neutrophil chemoattractant CXCL8/IL-8, MMP-9 further stimulates neutrophil recruitment; (**C**) whereas general inhibition of MMP-9 mixtures tempers inflammation, our results show that specific inhibition of the trimeric proteoform of MMP-9 further increases neutrophil chemotaxis, suggesting a role for MMP-9 trimers in tempering excessive neutrophil migration.

The process of neutrophil migration at early stages in infection or inflammation, i.e., from the bone marrow into the blood and through endothelia into challenged tissues (Figure 7A), is assisted by the actions of MMPs at several different levels and by different mechanisms [12,13,42]. In addition to their originally studied roles in extracellular matrix remodeling during cell migration, MMPs can also modulate chemotactic gradients by processing of chemokines or by cleaving key cell surface receptors and anchors involved in leukocyte rolling and transmigration [6]. Hence, specific inhibition of particular proteases (or combinations of proteases) during inflammation, represents an interesting approach for treatment of excessive inflammation. We here identified a bivalent carboxylate inhibitor, previously developed as a molecule to disrupt MMP-9 homodimerization in models for cancer cell migration [26], as a highly specific subnanomolar inhibitor for the stable trimeric proteoform of MMP-9 (IC_50_ = ±0.1 nM). In addition to trimeric MMP-9, the inhibitor was also potent at inhibiting MMP-2 (IC_50_ = 5 nM), MMP-3 (IC_50_ = 7.7 nM) and MMP-8 (IC_50_ = 14.5 nM), albeit at more than 50-fold higher doses. Similar inhibition of the monomeric proteoform of MMP-9 required at least a 500-fold higher concentration of the inhibitor (IC_50_ = 56 nM). Whereas we have previously shown that monomeric and trimeric MMP-9 are differentially inhibited by the natural inhibitors TIMP-1 [21] and α2M [22], the bivalent compound **7** is the first synthetic inhibitor to discriminate between monomeric and trimeric MMP-9.

For several reasons, MMP-9 is a key molecule in inflammation; (i) MMP-9 is pre-stored in secretory granules of neutrophils and is released upon neutrophil activation, free from natural inhibitors (TIMP-1) [12,48], (ii) MMP-9 is able to cleave the early neutrophil chemoattractant CXCL8/IL-8 and increase its potential 10-fold [43] (Figure 7B), (iii) together with MMP-2, MMP-9 cleaves mouse GCP-2/LIX, factually named CXCL6, influencing in vivo chemotaxis [12] and increasing neutrophil migration in IL-1β-induced peritonitis [42], (iv) immune cell-derived MMP-2 and MMP-9 are required for immune cell migration towards the central nervous system [14]. Furthermore, both MMP-3 [41] and MMP-8 [49] also contribute to neutrophil-recruitment, for example through CXCL6 activation. Hence, the application of compound **7** as an inhibitor for early chemotaxis seemed plausible. Unexpectedly, our results show that compound **7** increases neutrophil migration towards *E. coli*-derived LPS (Figure 7C) and opposes the effects of compound **5** which had lower MMP-specificity. Furthermore, in a mouse model for acute and systematic inflammation (mouse endotoxemia model), administration of compound **7** did not alter disease scores compared to compound **5**. This might point to a detrimental role for MMP-9 (of all proteoforms) during the early stages of endotoxin shock and be an indication that general, short-time, MMP-9 inhibition during the very early stages of endotoxin shock remains feasible. In contrast to the disease scores, the levels of MMP-9 in plasma and lungs from animals receiving compound **7** were increased which is indicative for extended inflammation and chemotaxis and in agreement with the results of the air pouch chemotaxis model. Since the changes in MMP-9 levels are not reflected in the disease scores, one might wonder whether the effects of MMP-9 trimers might be even more relevant at later time-points or in pathologies such as chronic inflammation. Unfortunately, most studies on the role of specific MMPs in inflammation are based on the use of genetic knock-out animals [50] or exogenous addition of in vitro processed chemokines. Therefore, many questions on how and where targeting of specific MMPs might be feasible in a therapeutic setting remain unanswered. Interestingly, in a study on the effect of pharmacological inhibition of MMP-9 on the outcome of acute myocardial infarction, an unexpected detrimental role of MMP-9 inhibition was also observed. MMP-9 inhibition with a triple-helical peptide inhibitor led to worsened cardiac function and a prolonged pro-inflammatory response, resulting in increased neutrophil and macrophage influx [51], and contradicting earlier studies in animals with genetic deletion of MMP-9 [52]. Overall, our results point towards a beneficial role for the trimeric proteoform of MMP-9 in neutrophil-mediated inflammation (in combination, or not, with inhibition of other MMPs including MMP-2, MMP-3 and MMP-8). Given the high amount of TIMP-free MMP-9 in neutrophils, our study mainly focused on neutrophils and acute inflammation, however, one might wonder what the effects of these inhibitors might be on other processes such as the induction of angiogenesis by M2 macrophages which secrete lower amounts of TIMP-1 [53].

In conclusion, neutrophil migration and resolution of inflammation is a complex balancing act between several physiological triggers, such chemoattractants, chemorepellents and even neutrophil reverse migration [54]. Although effects from off-target MMP-inhibition can never fully be excluded with pharmacological MMP inhibitors, administration of an inhibitor with high inhibitory activity against trimeric MMP-9, increased the presence of neutrophils at the site of an inflammatory trigger, suggesting a possible beneficial role for MMP-9 trimers in tempering excessive neutrophil migration or in resolution of inflammation. Furthermore, administration of the inhibitor at onset of endotoxin shock, worsened the early progression of the acute systematic inflammation in a mouse endotoxemia model. In addition, for the first time, we have identified a small molecule inhibitor with a high selectivity for the trimeric proteoform of MMP-9, which will allow further detailed research on the exact functions of specific MMP-9 proteoforms.

## Figures and Tables

**Table 1:** Inhibitory capacity of a monovalent and the corresponding bivalent carboxylate inhibitor on gelatinolysis by MMP-9 monomers and trimers.

MMP-9 Preparation/Substrate	MMP-9 Form	Monovalent Inhibitor (Compound 5)					Bivalent Inhibitor (Compound 7)				
		Model	*n*	IC_25_	IC_50_	IC_75_	Model	*n*	IC_25_	IC_50_	IC_75_
		(R^2^)		(CI) (nM)	(CI) (nM)	(CI) (nM)	(R^2^)		(CI) (nM)	(CI) (nM)	(CI) (nM)
Recombinant ^1^/gelatin degradation	mix ^4^	dose response	7	359	1407	5509	biphasic	9	28	182	627
		(0.98)		(325–396)	(1307–1517)	(4879–6260)	(0.97)		(19–38)	(158–207)	(539–740)
	mono	dose response	7	392	1516	5859	dose response	10	48	191	754
		(0.96)		(339–449)	(1369–1683)	(4951–7024)	(0.92)		(39–58)	(166–220)	(612–946)
	tri	dose response	7	526	2253	9660	biphasic	10	0.03	0.18	18
		(0.97)		(466–589)	(2058–2477)	(8228–NA)	(0.93)		(0.019–0.027)	(0.14–0.24)	(5–42)
Recombinant ^1^/peptide degradation ^2^	mix ^4^	dose response	4	228	726	2309	dose response	6	1.4	15	169
		(0.99)		(209–248)	(682–773)	(2097–2553)	(0.94)		(1.0–1.9)	(12–19)	(125–230)
	mono	dose response	4	232	718	2228	dose response	4	10.5	56	296
		(0.99)		(220–244)	(693–745)	(2109–2357)	(0.96)		(7.7–14.2)	(45–69)	(225–397)
	tri	dose response	4	237	724	2211	dose response	4	0.0017	0.10	6.0
		(0.99)		(223–252)	(693–757)	(2070–2366)	(0.82)		(0.0006–0.0046)	(0.05–0.18)	(3.1–12.5)
neutrophil-derived ^3^/gelatin degradation	mono	dose response	2	300	1154	4431	dose response	3	10	43	181
		(0.97)		(259–345)	(1026–1312)	(NA–NA)	(0.96)		(8–13)	(35–52)	(139–240)
	tri	dose response	2	479	4223	17212	biphasic	4	0.008	0.066	0.6
		(0.95)		(398–577)	(NA-NA)	(NA–NA)	(0.93)		(NA–0.013)	(0.05–0.09)	(0.4–1.1)

Concentration at which gelatinolysis is reduced by 25 (IC_25_), 50 (IC_50_) or 75 (IC_75_) percent. IC values are reported as best-fit value and 95% confidence interval (CI). R^2^ = goodness of curve fit. *n* = number of experiments. Concentrations are in nanomolars (nM). ^1^ human MMP-9, produced in Sf9 insect cells. ^2^ Fluorogenic peptide assay (OmniMMP/Mca-PLGL-Dpa-AR-NH2).^3^ MMP-9 isolated from human neutrophils. ^4^ MMP-9 mixture consisting of ± 70% monomers and 30% trimers, reflecting baseline biological conditions. NA; no data available due to out of range or low accuracy.

**Table 2:** Inhibitory capacity of monovalent and bivalent carboxylate inhibitors **5** and **7** on proteases with importance for acute inflammation.

Proteases	Assay Substrate	Monovalent Inhibitor (Compound 5)			Bivalent Inhibitor (Compound 7)		
		Model	*n*	IC_50_	Model	*n*	IC_50_
		(R^2^)		(CI) (nM)	(R^2^)		(CI) (nM)
MMP-9 monomers	Peptide ^1^	dose response	4	718	dose response	4	56
		(0.99)		(693–745)	(0.96)		(45–69)
MMP-9 trimers	Peptide ^1^	dose response	4	724	dose response	4	0.10
		(0.99)		(693–757)	(0.82)		(0.05–0.18)
neutrophil elastase	Peptide ^2^ and gelatin	NA	3	no inhibition ^†^	NA	3	no inhibition ^†^
TACE/ADAM17	Peptide ^3^	NA	2	no inhibition ^†^	NA	2	no inhibition ^†^
MMP-8/neutrophil collagenase	Peptide ^1^	dose response	5	331	dose response	5	14.5
		(0.94)		(288–385)	(0.96)		(12.5–16.7)
MMP-14/MT1-MMP	Peptide ^1^	dose response	4	14795	dose response	4	1076
		(0.91)		(12048–18437)	(0.92)		(885–1311)
MMP-7	Peptide ^1^	dose response	4	> 10000	dose response	4	>10000
		(NA)		(NA–NA)	(0.29)		(NA–NA)
MMP-3	Peptide ^1^	dose response	4	965	dose response	4	7.7
		(0.86)		(753–1225)	(0.96)		(6.4–9.1)
MMP-2	gelatin	dose response	5	1724	dose response	5	4.8
		(0.97)		(1529–1953)	(0.99)		(4.3–5.4)
	Peptide ^1^	dose response	6	1029	dose response	6	5.0
		(0.91)		(869–1238)	(0.97)		(4.4–5.6)

Half-maximal inhibitory concentration (IC_50_), reported as best-fit value and 95% confidence interval (CI). R^2^ = goodness of curve fit. *n* = number of experiments. Concentrations are in nanomolar (nM). ^†^ inhibition was tested up to an inhibitor concentration of 50 µM. ^1^ OmniMMP substrate peptide. ^2^ fluorogenic elastase substrate. ^3^ Mca-PLAQAV-Dpa-RSSSR-NH_2_ fluorogenic peptide. NA; no data available due to out of range, low accuracy or no inhibition.

## Supplementary Materials

The following are available online at https://www.mdpi.com/2073-4409/9/7/1634/s1, Figure S1: gelatin zymography analysis of human recombinant and human neutrophil-derived MMP-9 monomers and trimers, Figure S2: Flow cytometry gating strategy for cellular identification, Figure S3: Full images of Western-blot analysis.
