# Supplementary material for: Bivalent Inhibitor with Selectivity for Trimeric MMP-9 Amplifies Neutrophil Chemotaxis and Enables Functional Studies on MMP-9 Proteoforms
Source: Cells. 2020 Jul 7;9(7):1634. doi: 10.3390/cells9071634 (PMC7408547; doi:10.3390/cells9071634)
Supplement: Supplementary file 1 [file cells-09-01634-s001.pdf]

Article

Bivalent Inhibitor with Selectivity for Trimeric MMP-9 Amplifies Neutrophil Chemotaxis and Enables Functional Studies on MMP-9 Proteoforms.

Elisa Nuti [1], Armando Rossello [1], Doretta Cuffaro [1], Caterina Camodeca [1], Jens Van Bael [2], Dries van der Maat [2], Erik Martens [2], Pierre Fiten [2], Rafaela Vaz Sousa Pereira [2], Estefania Ugarte-Berzal [2], Mieke Gouwy [3], Ghislain Opdenakker [2], Jennifer Vandooren [2,*]

[1] Department of Pharmacy, University of Pisa, Via Bonanno 6, 56126 Pisa, Italy; elisa.nuti@farm.unipi.it (E.N.); armando.rossello@farm.unipi.it (A.R.); doretta.cuffaro@farm.unipi.it (D.C.); caterina.camodeca@unipi.it (C.C.)

[2] Laboratory of Immunobiology, Department of Microbiology, Immunology and Transplantation, Rega Institute for Medical Research, University of Leuven, KU Leuven, Herestraat 49 - bus 1044, B-3000 Leuven, Belgium; jens.vanbael@kuleuven.be (J.V.B.); driesvandermaat@hotmail.com (D.v.d.M.); erik.martens@kuleuven.be (E.M.); pierre.fiten@kuleuven.be (P.F.); rafaela.pereira@kuleuven.be (R.V.S.P.); estefania.ugarteberzal@kuleuven.be (E.U.-B.); ghislain.opdenakker@kuleuven.be (G.O.)

[3] Laboratory of Molecular Immunology, Department of Microbiology, Immunology and Transplantation, Rega Institute for Medical Research, University of Leuven, KU Leuven, Herestraat 49 - bus 1044, B-3000 Leuven, Belgium. mieke.gouwy@kuleuven.be (M.G.)

* Correspondence: jennifer.vandooren@kuleuven.be; Tel.: +32-16-32-22-95

Received: 9 June 2020; Accepted: 2 July 2020; Published: xx

Supplementary material

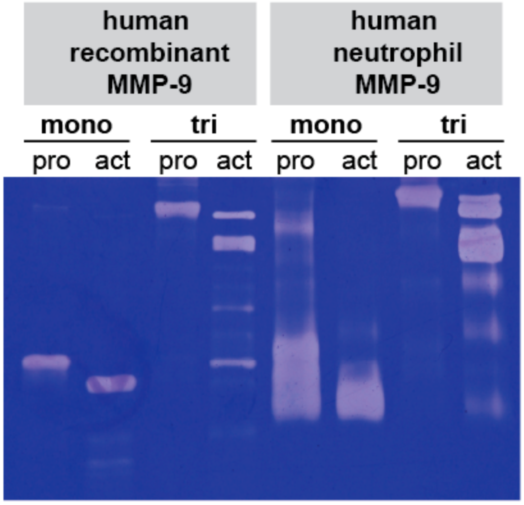

Figure 1. gelatin zymography analysis of human recombinant and human neutrophil-derived MMP-9 monomers and trimers. A shift in molecular weight is observed after activation with the catalytic domain of MMP-3.

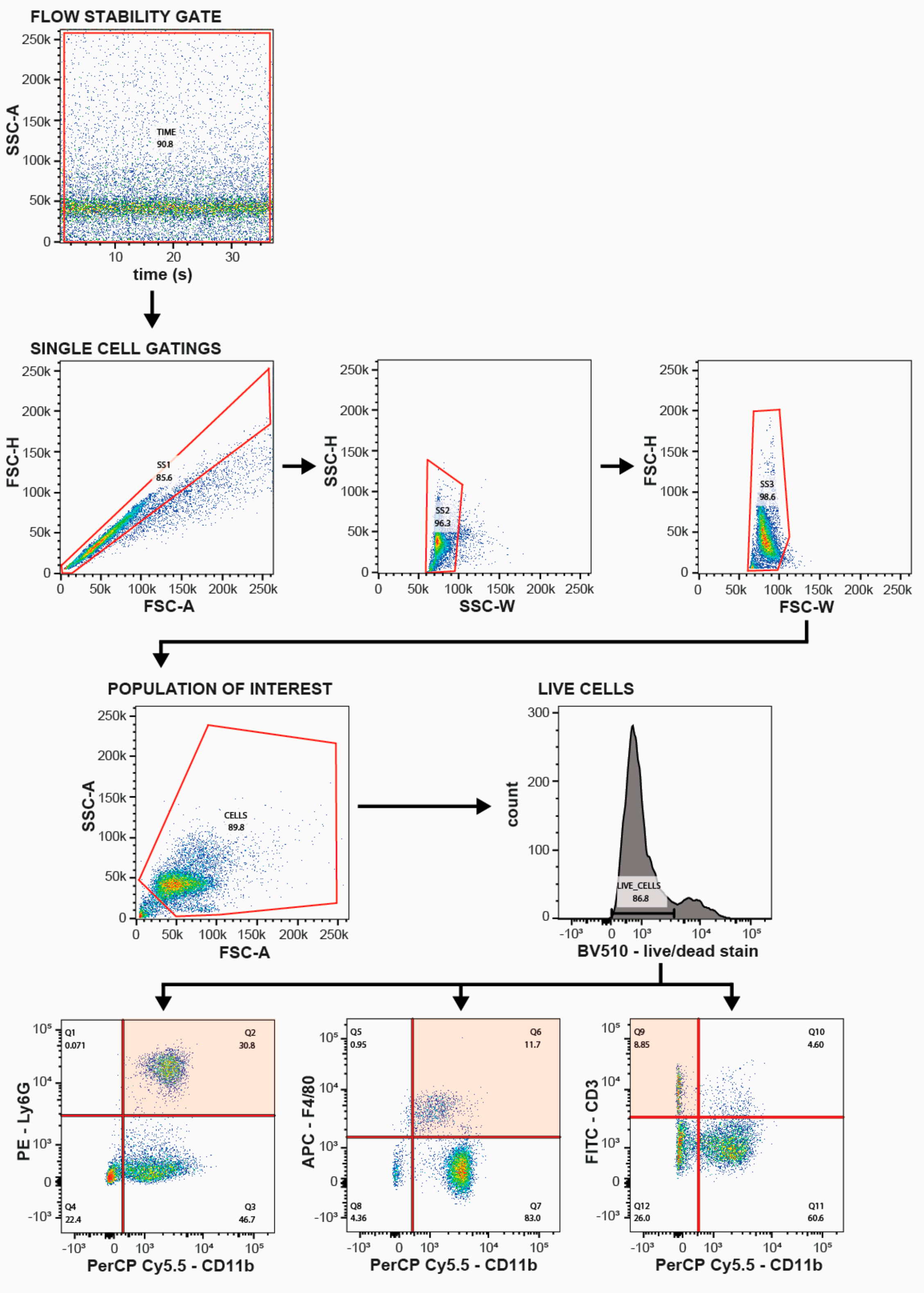

Figure 2. Flow cytometry gating strategy for cellular identification. Representative flow cytometry dot plots for analysis of CD11b$^+$Ly6G$^+$ neutrophils, CD11b$^+$F4/80$^+$ monocytes and CD11b$^-$CD3$^+$ T lymphocytes from mouse air pouches.

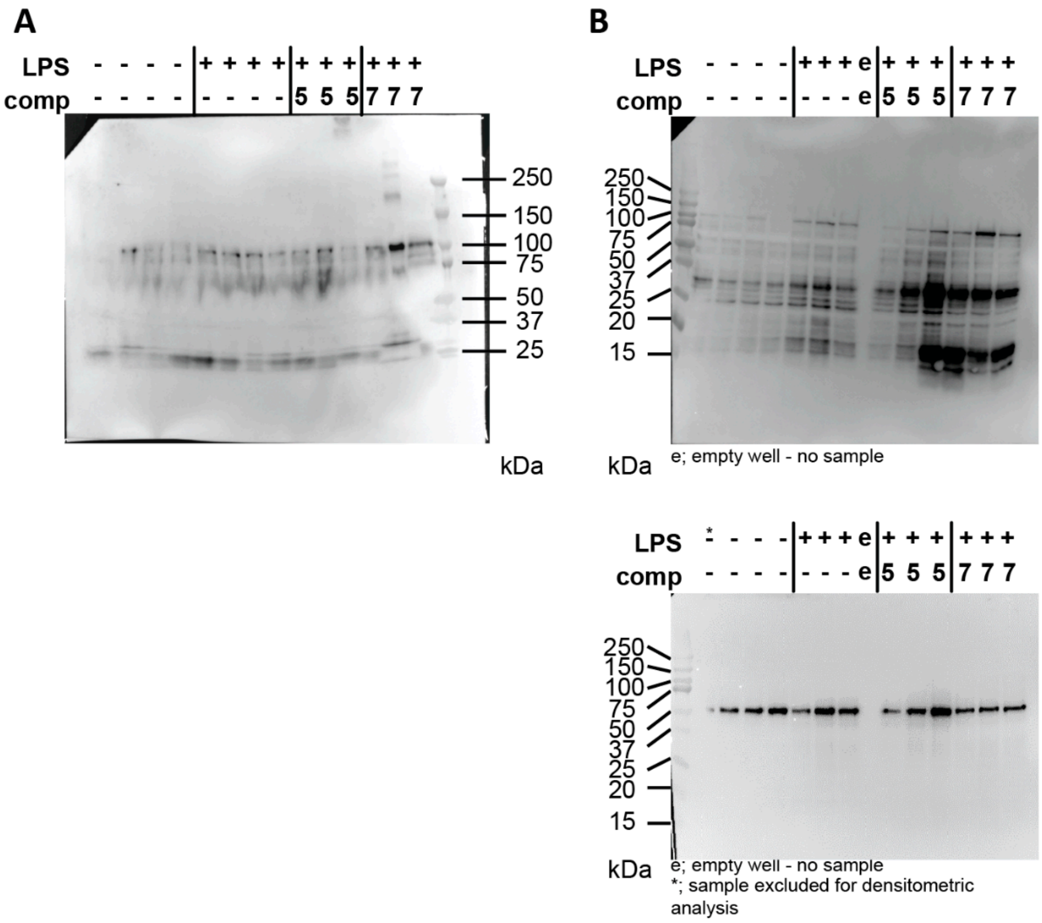

Figure 3. Full images of Western-blot analysis. (**A**) Western-blot analysis of mouse plasma 5 hours after LPS/compound administration. Signal detected with anti-MMP-9 antibody. Full image of Wester-blot analysis shown in Figure 6E. (**B**) Western-blot analysis of mouse lung extract, 5 hours after LPS/compound administration. Top panel; signal detected with anti-MMP-9 antibody. Bottom panel; signal detected with anti-tubulin antibody. Full images of Wester-blot analyses shown in **Figure 6F**. +/- signs indicate, respectively, the presence or absence of LPS (2 μg).
